# Biological Effects of Hydrogen Water on Subjects with NAFLD: A Randomized, Placebo-Controlled Trial

**DOI:** 10.3390/antiox11101935

**Published:** 2022-09-28

**Authors:** Branislav Kura, Maria Szantova, Tyler W. LeBaron, Viliam Mojto, Miroslav Barancik, Barbara Szeiffova Bacova, Barbora Kalocayova, Matus Sykora, Ludmila Okruhlicova, Narcisa Tribulova, Anna Gvozdjakova, Zuzana Sumbalova, Jarmila Kucharska, Xenia Faktorova, Martina Jakabovicova, Zuzana Durkovicová, Jan Macutek, Michaela Koscová, Jan Slezak

**Affiliations:** 1 Centre of Experimental Medicine, Institute for Heart Research, Slovak Academy of Sciences, 841 04 Bratislava, Slovakia; 2 3rd Department of Internal Medicine, Faculty of Medicine, Comenius University, 813 72 Bratislava, Slovakia; 3 Molecular Hydrogen Institute, Enoch, UT 84721, USA; 4 Department of Kinesiology and Outdoor Recreation, Southern Utah University, Cedar City, UT 84721, USA; 5 Pharmacobiochemical Laboratory of 3rd Medical Department, Medical Faculty, Comenius University Bratislava, 811 08 Bratislava, Slovakia; 6 Internal Clinic of Slovak Medical University, Hospital of St. Michael, 811 08 Bratislava, Slovakia; 7 Mathematical Institute, Slovak Academy of Sciences, 814 73 Bratislava, Slovakia; 8 Department of Mathematics, Faculty of Natural Sciences, Constantine the Philosopher University in Nitra, 949 01 Nitra, Slovakia

**Keywords:** inflammation, matrix metalloproteinases, molecular hydrogen, NAFLD, oxidative stress, ROS

## Abstract

Non-alcoholic fatty liver disease (NAFLD) is a liver pathology affecting around 25% of the population worldwide. Excess oxidative stress, inflammation and aberrant cellular signaling can lead to this hepatic dysfunction and eventual carcinoma. Molecular hydrogen has been recognized for its selective antioxidant properties and ability to attenuate inflammation and regulate cellular function. We administered hydrogen-rich water (HRW) to 30 subjects with NAFLD in a randomized, double-blinded, placebo-controlled manner for eight weeks. Phenotypically, we observed beneficial trends (*p* > 0.05) in decreased weight (≈1 kg) and body mass index in the HRW group. HRW was well-tolerated, with no significant changes in liver enzymes and a trend of improved lipid profile and reduced lactate dehydrogenase levels. HRW tended to non-significantly decrease levels of nuclear factor kappa B, heat shock protein 70 and matrix metalloproteinase-9. Interestingly, there was a mild, albeit non-significant, tendency of increased levels of 8-hydroxy-2’-deoxyguanosine and malondialdehyde in the HRW group. This mild increase may be indicative of the hormetic effects of molecular hydrogen that occurred prior to the significant clinical improvements reported in previous longer-term studies. The favorable trends in this study in conjunction with previous animal and clinical findings suggest that HRW may serve as an important adjuvant therapy for promoting and maintaining optimal health and wellness. Longer term studies focused on prevention, maintenance, or treatment of NAFLD and early stages of NASH are warranted.

## 1. Introduction

Non-alcoholic fatty liver disease (NAFLD) is a metabolic dysfunction of the liver with excess deposits of fat accumulation caused by factors other than alcohol. Approximately 25% of the world’s population have NAFLD, which makes it the most common hepatic pathology worldwide [1]. Left untreated, NAFLD can progress to nonalcoholic steatohepatitis (NASH), a pathological inflammatory condition of the liver with concomitant fibrosis, which can result in cirrhosis and hepatocarcinoma [2]. However, there is no simple cure for NAFLD, and its treatment generally relies on management and changes in dietary and lifestyle activities. Accordingly, preventing its occurrence or further progression by reducing excess fat accumulation, oxidative stress and inflammation may be a useful strategy [3]. 

Molecular hydrogen has emerged as a novel medical gas with antioxidant, anti-inflammatory effects, as demonstrated in studies involving animals and humans [4] and even in plants [5]. Hydrogen can be inhaled or simply dissolved in water to produce hydrogen-rich water (HRW). Ingestion of HRW leads to a peak plasma and breath H_2_ concentration in 5–15 min and returns to baseline in approximately 60 min [6]. Ingestion of HRW was shown to be comparable to sulfasalazine in a mouse model of colitis [7]. Furthermore, HRW prevented oxidative stress-induced liver fibrogenesis in mice [8] and activated peroxisome proliferator-activated receptor (PPAR) alpha and PPAR gamma expression in hepatocytes [9]. 

Clinical studies have demonstrated favorable effects of HRW in subjects with metabolic syndrome [10,11]. Similarly, a 28-day pilot study demonstrated that HRW reduced hepatic lipid accumulation in NAFLD subjects without significantly influencing lipid parameters [12]. The aim of this study was to analyze the effects of eight-week administration of HRW on body composition, lab chemistry profiles, oxidative stress and markers related to inflammation in subjects with NAFLD.

## 2. Materials and Methods

### 2.1. Test Subjects and Study Design

In total, 30 subjects (13 male, 17 female, average age 52.9 ± 10.6 years) with diagnosed NAFLD were included in the study and evaluated at baseline and at 8 weeks. The inclusion criteria for this experiment were steatosis according to USG (ultrasonography), or increased alanine transaminase (ALT), aspartate aminotransferase (AST), gamma-glutamyl transferase (GMT), overweight/obesity, and a questionnaire containing questions about lifestyle risk factors (20 q), together with excluding other etiology of the liver disease. Patients with currently treated cancer, actively treated rheumatological diseases by biological treatment, active tuberculosis, acute respiratory disease, or acute gastroenteritis were excluded from the study. The whole experimental group of patients with NAFLD was routinely treated with the same drugs (antihypertensive drugs, antidiabetics, hypolipidemics) without any change. None of the participants in the study had a partner or spouse who participated in the study. Written informed consent was obtained from all participants and the trial was conducted in accordance with the Declaration of Helsinki. The study was approved by the Regional Ethical Review Board in Bratislava, Slovak Republic (NCT 05325398). The baseline anthropometric data of the subjects are indicated in Table 1.

**Figure 1:**
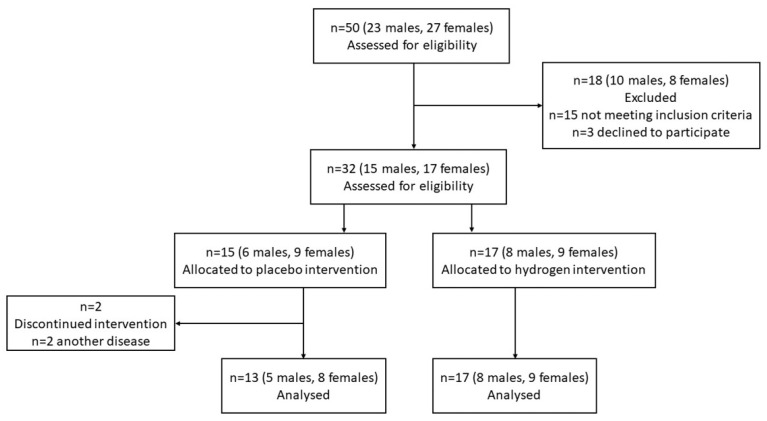
CONSORT flow diagram.

All selected patients were randomly divided into two groups in a double-blinded fashion where patients either consumed HRW (hydrogen group; *n* = 17) or non-HRW (placebo group; *n* = 13) (Figure 1). The randomization was performed by a computerized random number generator. The sequence was generated by the process of minimization by M.S. Patients in the HRW group received hydrogen-producing tablets with the ability to enrich regular water with molecular hydrogen by the aqueous reaction between elemental magnesium and organic acids (Drink HRW and Natural Wellness Now Health Products Inc., Vancouver, BC, Canada), as described previously [13]. Patients in the placebo group received tablets that were similar in appearance and ingredients (i.e., magnesium carbonate, citric acid, sodium bicarbonate, Inulin, Kollidon 30, sodium stearyl fumarate), where CO_2_ (g) was produced instead of H_2_ (g) (Drink HRW and Natural Wellness Now Health Products Inc., Vancouver, BC, Canada). All groups were instructed to dissolve one tablet in 330 mL of water, wait for dissolving and drink the produced HRW (>4 mg/L H_2_) immediately, three times per day for two months. Blood plasma was collected in a sodium citrate solution in the morning hours (before 8 a.m.) after the previous night fasting at the start and the end of the experiment. Collected plasma was separated by centrifugation (3000× *g* for 10 min at 4 °C) and immediately stored at −80 °C until analysis.

### 2.2. Clinical Biochemical/Hematological Parameters

All biochemical/hematological parameters (albumin, alkaline phosphatase, alanine transferase, aspartate aminotransferase, C-reactive protein, total cholesterol, high density lipoprotein, triglycerides) were measured by SYNLAB, Bratislava. Chemistry Analyzer AU5812 (Beckman Coulter, Brea, CA, USA) was used for assessment biochemical parameters with IFCC method for ALT, AST. ALP, colorimetric method, BCG for assessment albumin. For lipid parameters (cholesterol, HDL- cholesterol, LDL-cholesterol, triglycerides), enzymatic CHOD-PAP and colorimetric test were used. For determination CRP immunoturbidimetric method was used. 

### 2.3. Markers of Oxidative Stress

#### 2.3.1. Malondialdehyde Measurement

Malondialdehyde (MDA) analysis was performed using TBARS Assay Kit (item no. 700870, Cayman chemicals, Ann Arbor, MI, USA) according to the manufacturer’s instructions. Briefly, 100 μL of blood plasma was mixed with the Color Reagent solution composed of acetic acid, sodium hydroxide and thiobarbituric acid. This mixture was boiled for 1 h and then immediately placed on ice for 10 min. After centrifugation (1600× *g* for 10 min at 4 °C) the color intensity of the reaction product (MDA) was measured at 530 nm (Synergy H1 microplate reader, Biotek, Santa Clara, CA, USA). The malondialdehyde was used as a standard.

#### 2.3.2. 8-hydroxydeoxyguanosine Measurement

8-hydroxydeoxyguanosine (8-OHdG) was measured by the enzyme-linked immunosorbent assay (ELISA) method using an 8-OHdG ELISA kit (Elabscience, Houston, TX, USA; E-EL-0028) according to the manufacturer´s instructions. Briefly, 50 μL of blood plasma was mixed with 50 μL of Biotinylated Detection Ab working solution specific to 8-OHdG and incubated for 45 min at 37 °C. The solution was removed from the wells and washed three times. The same procedure was followed with 100 μL of horseradish peroxidase (HRP) conjugate working solution and 90 μL of substrate reaction solution (incubation time was 30 and 15 min, respectively, at 37 °C). The reaction was terminated by adding 50 μL of stop solution. The plate was measured at 450 nm (Synergy H1 microplate reader, Biotek, Santa Clara, CA, USA). 

### 2.4. Lactate Dehydrogenase Assay

The activity of lactate dehydrogenase (LDH) analysis was performed with a lactate dehydrogenase activity assay kit (Sigma-Aldrich, Saint Louis, MO, USA) according to the manufacturer´s recommendation. 2 μL of blood plasma was mixed with 48 μL of LDH assay buffer and with master reaction mix (composed of 48 μL LDH assay buffer and 2 μL LDH substrate mix). Standard NADH was used. This mix of solutions and samples was measured every 2–3 min at 450 nm (Synergy H1 microplate reader, Biotek, Santa Clara, CA, USA) at 37 °C until the value of the most active sample was greater than the value of the highest standard.

### 2.5. Western Blot

Protein analysis was carried out according to our previous study (Kura et al., 2019). Blood plasma samples treated with Laemmli buffer were applied on 12.5% sodium dodecyl sulfate-polyacrylamide gels (SDS–PAGE), separated by electrophoresis (120 V) and transferred to nitrocellulose membranes. Transferred proteins were visualized using Ponceau S solution (Sigma, St. Louis, MO, USA). Membrane blots were washed with TBS (1M Tris, 5 M NaCl in H_2_O, pH 7.4, Tween-20), blocked with 5% BSA in TBS for 4 h, and incubated with the appropriate primary antibodies at the specific dilutions referred to in Supplementary Table S1. The membrane was then washed and the primary antibodies were detected with goat anti-goat IgG, anti-mouse, or goat anti-rabbit IgG conjugated to HRP for 1 h. The membrane was then washed again with TBS solution, and then the solutions luminol (chromophore) and coumaric acid and hydrogen peroxide were applied to induce a chemiluminescent reaction. The chemiluminescent reaction was detected in an Amersham Imager 600 instrument (GE Healthcare Bio-Sciences AB, Danderyd, Sweden). All data obtained from Western blot were calculated with ImageJ software version 1.8.0_172.

### 2.6. Measurement of Matrix Metalloproteinases Activities by Gelatin Zymography

The activities of matrix metalloproteinases (MMPs)-2 and MMP-9 were evaluated using zymography in 10% polyacrylamide gels containing gelatin (2 mg/mL) as a substrate. The diluted plasma samples were prepared in Laemmli buffer without 2-mercaptoethanol and loaded onto gels without denaturation. After electrophoresis, the gels were washed with 50 mM Tris-HCl (pH 7.4), containing 2.5% Triton X-100 and then incubated overnight at 37 °C in a substrate buffer containing 50 mM Tris-HCl, 10 mM CaCl_2_ and 1.25% Triton X-100, pH 7.4. After incubation, the gels were stained with 1% Coomassie Brilliant Blue G-250 and then destained with 40% methanol and 10% acetic acid. The gelatinolytic activities of the MMP-2 and MMP-9 were detected as transparent bands against a dark blue background. 

### 2.7. Statistical Analysis

The sample size of subjects recruited was determined according to availability, willingness and the statistically minimal sample size needed. Statistical analyzes were performed by JM using an R statistical software environment (www.r-project.org, accessed on 11 July 2022). All significance levels were set at α = 0.05. Normality of the data was tested using the Shapiro–Wilk test. If the test rejected normality, the Mann–Whitney test was applied; if not, two-sample t-tests were used.

## 3. Results

### 3.1. Subjects

This study was performed in a randomized double-blinded, placebo-controlled fashion. All selected patients (13 males and 17 females) were from the Bratislava region (Slovak Republic). The average age was 53.23 (±9.13) years for placebo and 52.65 (±11.9) for the hydrogen group. Almost all patients had obesity, with an average value of body mass index (BMI) of 32.8 (±3.37) in placebo and 35.52 (±4.03) in the hydrogen group. All recruited subjects completed the trial without adverse events. For both groups, the average blood pressure was in a normal range.

Two months consumption of HRW had a positive trend in almost all measured basic characteristics of patients: weight (decreased), BMI (decreased), systolic blood pressure (decreased). However, these measurements did not show a statistical significance (*p* > 0.05) (Table 2).

### 3.2. Consumption of HRW Improved Plasma Biomarkers

Biochemical parameters indicative of lipid levels and liver functions as well as blood analysis were measured with standard biomedical and hematological methods in a clinical biochemical/hematological lab. For each group, we compared the individual biomarkers of their baseline values to their values after 8 weeks follow up (Table 3).

In both groups, there was a similar nonsignificant decrease in AST and ALT (*p* > 0.05). On the other hand, both groups had a mild increase in CRP, ALB, ALP, TG, TC, HDL, LDL and the TG/HDL ratio (*p* > 0.05). These non-significant increases were the greatest in the HRW group, except for CRP, TG, LDL and the TG/HDL ratio, which increased more in the placebo group (*p* > 0.05). In contrast, LDH levels tended to increase (2.63%) in the placebo group and decrease (5.98%) in the HRW group (*p* > 0.05).

### 3.3. Biomarkers of Redox Status

**Figure 2:**
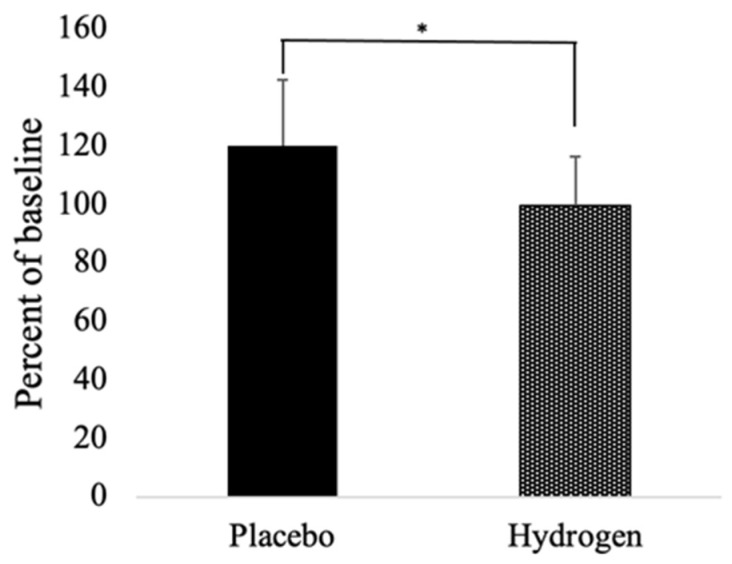
Relative percent change from baseline to follow up of SOD1. *n* = 13 for placebo group, *n* = 17 for hydrogen group, * *p* < 0.05.

As seen in Table 3, the levels of MDA tended to decrease in the placebo group, but increase in the HRW group, which difference between the two groups reached statistical significance (*p* < 0.05). However, the post-MDA level in the HRW group was about the same as the baseline level in the placebo group, 3.13 ± 1.60 and 3.11 ± 1.53, respectively. Both groups had non-significant increase in 8-OHdG, with a tendency to have a greater increase in the HRW group. We also monitored levels of Cu/Zn superoxide dismutase (SOD1). As seen in Figure 2, the levels of SOD1 increased (≈20 ± 16.71%) in the placebo group but remained unchanged (0.04 ± 22.61% increase) in the HRW group. The difference between the groups reached statistical significance (*p* < 0.05).

### 3.4. Inflammatory Markers and Heat Shock Proteins

**Figure 3:**
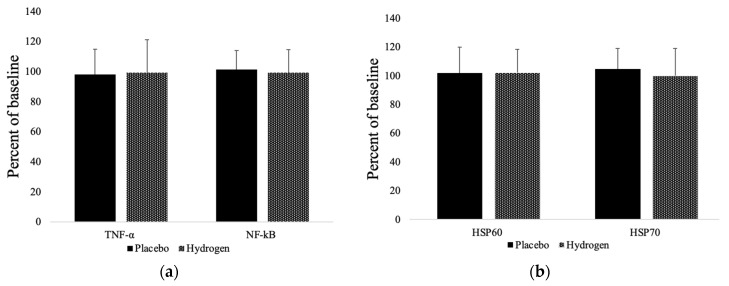
Markers of inflammation (**a**) and levels of heat shock proteins (**b**). HSP60 = heat shock protein 60; HSP70 = heat shock protein 70; TNF-α = tumor necrosis factor alpha; NF-κB = nuclear factor kappa B. *n* = 13 for placebo group, *n* = 17 for hydrogen group, *p* < 0.05.

As illustrated in Figure 3, the inflammatory marker, tumor necrosis factor-alpha (TNF-α), did not significantly change in either group from baseline compared to follow up (placebo 98.20 ± 16.55%; HRW 99.34 ± 21.71%). However, nuclear factor kappa B (NF-κB) had a non-significant tendency to increase in the placebo group (101.23 ± 12.62%) and decrease in the HRW group (99.41 ± 15.15%). Similarly, heat shock protein-60 (HSP60) and HSP70 were unchanged in either group. However, HSP70 mildly increased in the placebo (104.60 ± 14.24%) and decreased in the HRW group (99.90 ± 19.14%), whereas HSP60 tended to increase in both groups (placebo 101.92 ± 18.06%; HRW 102.37 ± 16.02%).

### 3.5. Matrix Metalloproteinases

**Figure 4:**
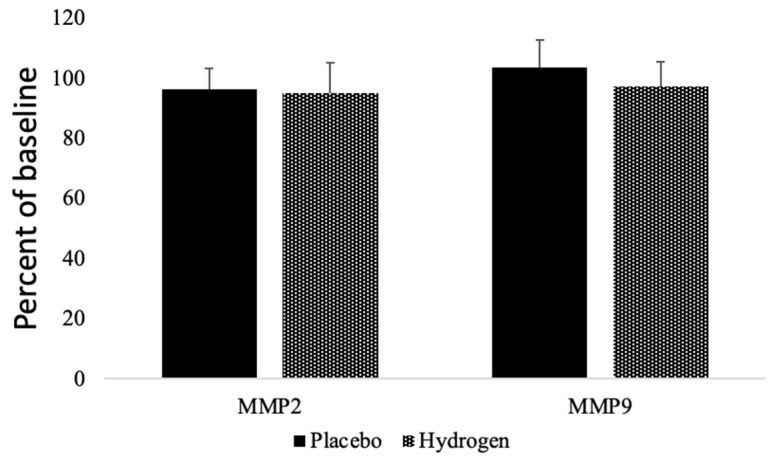
Effects of HRW on Matrix metalloproteinases. MMP = matrix metalloproteinases. *n* = 13 for placebo group, *n* = 17 for hydrogen group, *p* < 0.05.

We found that the plasma levels of matrix metalloproteinases (MMPs), MMP2 had a non-significant trend to decrease in both the placebo (96.15 ± 7.14%) and in the HRW (95.07 ± 10.09%) groups. Moreover, MMP9 tended to increase in the placebo group (103.67 ± 8.89%) and decrease in the HRW group (97.12 ± 8.36%), but the difference remained statistically insignificant (Figure 4).

## 4. Discussion

In this randomized, double-blinded, placebo-controlled study, we investigated the effects of high-dose HRW (i.e., >12 mg/day) in subjects with NAFLD for 8 weeks. There were no adverse events with HRW intervention as indicated by non-significant changes in liver enzymes. Moreover, the frequent consumption, three times per day, of HRW was well-tolerated by subjects.

In the two-month study, there was a trend of increased weight gain in the placebo group (≈0.23 kg). In contrast, in the HRW group, the opposite trend occurred, with an average body mass reduction of ≈1 kg. Although the change in weight was statistically insignificant, there was a statistically significant decrease in BMI in the HRW group compared to the placebo group. These improvements in weight and BMI may have clinically meaningful implications, especially given the short duration of our study. These results are corroborated with our previous finding that 24 weeks of high-concentration HRW consumption in subjects with metabolic syndrome significantly reduced body weight and BMI [10].

We found a positive trend in the HRW group of reduced systolic blood pressure (≈2 mm Hg). However, this change did not reach statistical significance. Perhaps this is due to the short duration of the study and relatively normal blood pressure values of the subjects. For example, diastolic blood pressure was below 80 mm Hg and systolic blood pressure was on the low end of pre-hypertensive criteria. However, although a 2 mm Hg reduction in systolic blood pressure is statistically insignificant, it may have clinical relevance [14]. Indeed, a meta-analysis found that a 2-mm Hg decrease in resting systolic blood pressure was associated with reductions in mortality of 4% in coronary heart disease, 6% from stroke and 3% from all causes [15].

We also investigated the effects of HRW on various biomarkers of liver function and cholesterol and observed beneficial trends in these areas. However, in contrast to other studies, the changes in these parameters remained insignificant, likely attributable to either (i) the values already being in homeostasis/range, or (ii) the shorter duration of the study. Our previous six-month investigation in subjects with metabolic syndrome showed improvements in many of these parameters [10], which corresponded to other clinical reports [11,16,17]. For example, HRW had an important trend of increasing HDL cholesterol compared to the placebo group. Moreover, HRW attenuated the rise in TG levels that occurred over the two months. These effects on TG and HDL resulted in a significantly better TG/HDL ratio in the HRW group compared to the placebo group (i.e., 3.64 vs. 4.25, respectively). That is, in the placebo group there was an 11.2% increase compared to only a 0.55% increase in the HRW group. The higher the TG/HDL ratio, the greater the risk for development of coronary disease [18], which also serves as an independent predictor for all-cause mortality [19]. Despite these favorable trends, the absolute changes were not as large compared to previous studies where baseline cholesterol levels were more significantly elevated [11,12,16,17]. In our study, total cholesterol was still below 200 mg/dL, which, for subjects of similar age, large prospective cohort studies suggest that having a TC level lower than 200 mg/dL is associated with increased mortality [20,21]. Since cholesterol levels and health may follow an inverse U-curve association [22], it would be interesting to test the effects of HRW administration on those with higher cholesterol, e.g., >250 mg/dL.

Molecular hydrogen has been reported to exert antioxidant effects in cell, animal and human clinical studies [4]. Compared to baseline, SOD1 levels slightly increased in the placebo group and remained the same in the HRW group. Interestingly, we observed a mild non-significant increase in MDA (≈17.2%) and in 8-OHdG (≈8.3%), which are markers of oxidative stress in the HRW group. These increases were significantly less than the increase induced following an intense exercise bout. For example, following a self-paced 21 km run at 77% VO_2_ peak in trained male runners, MDA increased by ≈11.5% [23] and 60-min running at a similar intensity increased urinary 8-OHdG by ≈276% [24]. These values should be compared to the several hundred percent increases often reported in chronic diseases and pathological conditions [25]. Moreover, unlike with exercise or molecular hydrogen, the oxidative stress in the diseased states remain chronically elevated, which results in antioxidant depletion and cellular injury. We found that levels of LDH were reduced by ≈6% following HRW administration, which may indicate a protective effect as previously demonstrated [26]. 

In addition to its known antioxidant effects, molecular hydrogen has also been shown to exert selective anti-inflammatory effects [4]. However, in contrast to previous studies our results show no changes in TNF-α, with only a slight tendency of decreased NF-κB by HRW. Heat shock proteins (HSP) can be upregulated by inflammation; accordingly, we observed no changes in either HSP60 or HSP70. However, HSP70 followed a similar trend as NF-κB levels in that they tended to increase in the placebo group and decrease in the HRW group. At the same time, we found that MMP-2 and MMP-9, which are regulated by inflammation, were also not significantly altered. However, MMP-9 had a modest tendency to increase in the placebo group and decrease in the HRW group. This is in line with previous research demonstrating that HRW potently reduced smooth muscle cell migration by inhibiting MMP-9 and MMP-2 [27]. The serum expression of MMPs is correlated with the serum levels of markers of liver damage [28], which were not significantly altered in either group. This raises the possibility that the null effects of HRW on MMPs could be due to their lower expression in the NAFLD subjects compared to the studies in which MMPs were significantly upregulated by a greater stimulus [29]. 

HRW has shown promise in attenuating many disease pathologies, including COVID-19 [30,31], due to its antioxidant, anti-inflammatory and anti-apoptotic effects [32]. In an animal model of diet-induced NAFLD, HRW effectively improved body composition and decreased lipid accumulation in the liver [33]. These benefits were at least partly mediated by HRW-induced upregulation in acetyl CoA oxidase, levels of adiponectin, reduced expression of CD36 and the inflammatory markers TNF-α and IL-6 [33]. This is in line with other studies demonstrating beneficial effects of HRW in NAFLD in animal models [9,34]. Furthermore, a 28-day double-blinded, placebo-controlled, crossover trial in 12 subjects with NAFLD revealed that HRW significantly attenuated liver fat accumulation as assessed by dual-echo MRI [11]. However, similar to our study, there were no statistically significant changes on metabolic profiles. 

We previously demonstrated in a double-blinded, placebo-controlled study of 60 subjects with metabolic syndrome that six-month administration of high-concentration HRW significantly improved body composition, lipid profiles, oxidative stress and inflammation [10]. These favorable effects were often greater than those reported in previous clinical studies of metabolic syndrome [11,12,16,17]. The reasons for the differences may be attributed to the shorter duration and lower dose of HRW in those studies. These same reasons may also account for the lack of statistically significant changes in our NAFLD study. Moreover, we were unable to track subject compliance to determine how tightly the protocol of using the HRW-producing tablets was followed. Additionally, HRW administration may have improved other biomarkers that we did not examine (e.g., CD36, PPAR expression, adiponectin, fibroblast growth factor-21, insulin sensitivity, etc.), which were improved in previous studies [4]. Furthermore, it should not be expected that HRW would significantly influence biomarkers that are already within the range of normal homeostasis. 

Importantly, although not statistically significant in our study, a hydrogen-induced increase in markers of oxidative stress has been previously reported [35]. Indeed, several studies show that the therapeutic benefits of H_2_ are also correlated with slightly increased levels of MDA [36], even in the sham group [37]. Similarly, we demonstrated that H_2_ prevented irradiation-induced increases in plasma levels of TNF-α and MDA [38]. However, when H_2_ was administered alone, TNF-α initially increased above control and then decreased and remained below both the irradiated group and the non-irradiated control, whereas MDA tended to initially decrease then increase [38]. This demonstrates that although H_2_ primarily reduces MDA, sometimes its therapeutic effects are associated with transiently increased levels of MDA. Similarly, some of the benefits of H_2_ in plants are also mediated by increases in ROS production [39]. Additionally, pretreatment of SH-SY5Y cells with H_2_ transiently increased mitochondrial superoxide production [40]. This was followed by an upregulation of the antioxidant enzymes via induction of the nuclear factor erythroid 2-related factor 2 (Nrf2) pathway, which resulted in cytoprotection from H_2_O_2_ induced oxidative stress [40]. The induction of the Keap1-Nrf2 pathway plays an important role in the protective effects of molecular hydrogen [4]. 

Our results correspond with the proposed hormesis model underlying the mechanistic mode of action for molecular hydrogen [41]. This may also be true for the discrepant changes in inflammatory markers and heat shock proteins, which H_2_ may increase or decrease depending on the time of testing [35,41,42]. It was recently shown that H_2_ induces the mitochondrial unfolded protein response, which can subsequently improve mitochondrial function and cellular regeneration [43]. The mitochondria and other redox-active heme prosthetic groups may be important targets for molecular hydrogen [44]. Accordingly, the actions of hydrogen might transiently increase various biomarkers of cellular stress similar to the hormetic effects of exercise, followed by their decrease and subsequent improvements in cellular function and clinical parameters [41]. However, additional and/or longer-term studies are needed to confirm a hormetic effect by determining if more favorable biological effects occur as are seen in other H_2_ studies.

## 5. Conclusions

In conclusion, our study adds to the growing body of literature that molecular hydrogen has favorable biological effects. However, in our study, most of the observed trends either did not reach statistical significance and/or, if they did, their clinical implications are not clear. Nevertheless, since ingestion of HRW is both simple and safe, there are practical applications for its daily use. Moreover, unlike pharmaceuticals but similar to exercise, it is likely that daily and long-term consumption of HRW is required to elicit optimal clinical effects. Significant changes in short periods, although reported, may be unlikely for most people and HRW should serve as an adjuvant wellness therapy of habit as opposed to a primary medicine for treatment. In short, although not as significant as other research, our preliminary results, in conjunction with previous studies, indicate that oral ingestion of HRW may provide beneficial effects for NAFLD and that H_2_ may act as a mild hormetic effector in eliciting these biological benefits, similarly to exercise. Longer-term studies are needed to determine if hormesis is involved, and studies focused on the prevention, maintenance and treatment of NAFLD and early stages of NASH are warranted.

## Figures and Tables

**Table 1:** Baseline characteristics of subjects.

Baseline Characteristics	Placebo	Hydrogen	*p*-Value
Age (years)	53.23 ± 9.13	52.65 ± 11.9	>0.1
Height (cm)	170.46 ± 10.41	168.94 ± 11.08	>0.1
Weight (kg)	95.77 ± 14.21	101.35 ± 15.14	>0.1
Body mass index (BMI)	32.8 ± 3.37	35.52 ± 4.03	>0.1
Systolic blood pressure	125.38 ± 11.98	128.47 ± 9.37	>0.1
Diastolic blood pressure	77.69 ± 8.32	77.94 ± 6.86	>0.1
Heart rate (beats/min)	73.31 ± 8.22	69.65 ± 8.96	>0.1

**Table 2:** Changes in anthropometric characteristics from baseline to 8-week follow up.

Characteristics	Placebo		Hydrogen	
	Before	After	Before	After
Weight	95.77 ± 14.21	96.00 ± 14.98	101.35 ± 15.14	100.26 ± 15.66
BMI	32.8 ± 3.37	32.91 ± 3.03	35.52 ± 4.03	35.16 ± 4.33 *
Systolic bp	125.38 ± 11.98	126.15 ± 11.93	128.47 ± 9.37	126.18 ± 9.28
Diastolic bp	77.69 ± 8.32	74.62 ± 7.76	77.94 ± 6.86	77.06 ± 4.70
Pulse	73.31 ± 8.22	71.64 ± 6.05	69.65 ± 8.96	71.29 ± 7.28

* Statistical significance *p* < 0.05.

**Table 3:** Plasma biochemical markers from baseline and after 8-week intervention.

Marker	Placebo		Hydrogen	
	Baseline	Follow Up	Baseline	Follow Up
Albumin (ALB) (g/L)	43.22 ± 2.6	44.47 ± 2.90	42.68 ± 2.36	44.58 ± 2.04
Alkaline phosphatase (ALP) (ukat/L)	1.07 ± 0.33	1.09 ± 0.37	1.28 ± 0.53	1.32 ± 0.55
Alanine transferase (ALT) (ukat/L)	0.69 ± 0.34	0.58 ± 0.24	0.77 ± 0.48	0.75 ± 0.30
Aspartate aminotransferase (AST) (ukat/L)	0.57 ± 0.22	0.48 ± 0.14	0.62 ±0.41	0.53 ± 0.22
C-reactive protein (CRP) (mg/L)	3.26 ± 1.50	4.49 ± 2.50	4.52 ± 3.55	4.57 ± 3.20
MDA (µM/µL)	3.11 ± 1.53	2.34 ± 0.60	2.67 ± 0.82	3.13 ± 1.60 *
8-OHdG (ng/mL)	26.93 ± 8.70	27.87 ± 7.67	24.36 ± 9.51	26.39 ± 9.72
LDH (mU/mL)	71.23 ±32.50	73.10 ± 39.16	68.53 ± 28.91	64.43 ± 32.82
Total cholesterol (TC) (mg/dL)	179.34 ± 31.60	187.76 ± 41.06	180.32 ± 39.28	190.06 ± 38.85
High density lipoprotein (HDL) (mg/dL)	44.50 ± 9.29	46.05 ± 8.70	43.38 ± 4.19	46.52 ± 3.96
Low density lipoprotein (LDL) (mg/dL)	114.34 ± 24.40	119.91 ± 30.70	116.33 ± 31.21	120.54 ± 30.63
Triglycerides (TG) (mg/dL)	170.19 ± 58.59	195.60 ± 69.62	156.88 ± 39.12	169.45 ± 74.47
TG/HDL ratio	3.82 ± 6.31	4.25 ± 8.00	3.62 ± 9.33	3.64 ± 18.80

* Statistical significance *p* < 0.05.

## Data Availability

The data is contained within the paper and the Supplementary Materials.

## Supplementary Materials

The following supporting information can be downloaded at: https://www.mdpi.com/article/10.3390/antiox11101935/s1. Table S1. Antibodies and dilutions used in Western blot. ^a^ Santa Cruz biotechnology, Dallas, TX, USA; sc-1350; ^b^ Cell Signaling, Beverly, MA, USA; 7074S.

## References

[1] Younossi Z.M., Koenig A.B., Abdelatif D., Fazel Y., Henry L., Wymer M. (2016). Global epidemiology of nonalcoholic fatty liver dis-ease-Meta-analytic assessment of prevalence, incidence, and outcomes. Hepatology.

[2] Buzzetti E., Pinzani M., Tsochatzis E.A. (2016). The multiple-hit pathogenesis of non-alcoholic fatty liver disease (NAFLD). Metabolism.

[3] Soret P.-A., Magusto J., Housset C., Gautheron J. (2020). In Vitro and In Vivo Models of Non-Alcoholic Fatty Liver Disease: A Critical Appraisal. J. Clin. Med..

[4] LeBaron T.W., Kura B., Kalocayova B., Tribulova N., Slezak J. (2019). A New Approach for the Prevention and Treatment of Cardio-vascular Disorders. Molecular Hydrogen Significantly Reduces the Effects of Oxidative Stress. Molecules.

[5] Hancock J.T., LeBaron T.W., May J., Thomas A., Russell G. (2021). Molecular Hydrogen: A new treatment for plants in the UK?. Plants.

[6] Ito M., Hirayama M., Yamai K., Goto S., Ito M., Ichihara M., Ohno K. (2012). Drinking hydrogen water and intermittent hydrogen gas exposure, but not lactulose or continuous hydrogen gas exposure, prevent 6-hydorxydopamine-induced Parkinson’s disease in rats. Med. Gas Res..

[7] LeBaron T.W., Asgharzadeh F., Khazei M., Kura B., Tarnava A., Slezak J. (2021). Molecular hydrogen is comparable to sulfasalazine as a treatment for DSS-induced colitis in mice. EXCLI J..

[8] Koyama Y., Taura K., Hatano E., Tanabe K., Yamamoto G., Nakamura K., Yamanaka K., Kitamura K., Narita M., Nagata H. (2013). Effects of oral intake of hydrogen water on liver fibrogenesis in mice. Hepatol. Res..

[9] Zhai X., Chen X., Lu J., Zhang Y., Sun X., Huang Q., Wang Q. (2017). Hydrogen-rich saline improves nonalcoholic fatty liver disease by alleviating oxidative stress and activating hepatic PPARalpha and PPARgamma. Mol. Med. Rep..

[10] LeBaron T.W., Singh R.B., Fatima G., Kartikey K., Sharma J.P., Ostojic S.M., Gvozdjakova A., Kura B., Noda M., Mojto V. (2020). The Effects of 24-Week, High-Concentration Hydrogen-Rich Water on Body Composition, Blood Lipid Profiles and Inflammation Biomarkers in Men and Women with Metabolic Syndrome: A Randomized Controlled Trial. Diabetes Metab. Syndr. Obes. Targets Ther..

[11] Nakao A., Toyoda Y., Sharma P., Evans M., Guthrie N. (2010). Effectiveness of Hydrogen Rich Water on Antioxidant Status of Subjects with Potential Metabolic Syndrome—An Open Label Pilot Study. J. Clin. Biochem. Nutr..

[12] Korovljev D., Stajer V., Ostojic J., LeBaron T.W., Ostojic S.M. (2019). Hydrogen-rich water reduces liver fat accumulation and improves liver enzyme profiles in patients with non-alcoholic fatty liver disease: A randomized controlled pilot trial. Clin. Res. Hepatol. Gastroenterol..

[13] LeBaron T.W., Larson A.J., Ohta S., Mikami T., Barlow J., Bulloch J., DeBeliso M. (2019). Acute Supplementation with Molecular Hydrogen Benefits Submaximal Exercise Indices. Randomized, Double-Blinded, Placebo-Controlled Crossover Pilot Study. J. Lifestyle Med..

[14] Cook N.R., Cohen J., Hebert P.R., O Taylor J., Hennekens C.H. (1995). Implications of small reductions in diastolic blood pressure for primary prevention. Arch. Intern. Med..

[15] Da G.A.K., Kelley K.A., Tran Z. (2001). Aerobic Exercise and Resting Blood Pressure: A Meta-Analytic Review of Randomized, Controlled Trials. Prev. Cardiol..

[16] Song G., Li M., Sang H., Zhang L., Li X., Yao S., Yu Y., Zong C., Xue Y., Qin S. (2013). Hydrogen-rich water decreases serum LDL-cholesterol levels and improves HDL function in patients with potential metabolic syndrome. J. Lipid Res..

[17] Kajiyama S., Hasegawa G., Asano M., Hosoda H., Fukui M., Nakamura N., Kitawaki J., Imai S., Nakano K., Ohta M. (2008). Supplementation of hydrogen-rich water im-proves lipid and glucose metabolism in patients with type 2 diabetes or impaired glucose tolerance. Nutr. Res..

[18] da Luz P.L., Favarato D., Faria-Neto J.R., Lemos P., Chagas A.C.P. (2008). High ratio of triglycerides to HDL-cholesterol predicts extensive coronary disease. Clinics.

[19] Sultani R., Tong D.C., Peverelle M., Lee Y.S., Baradi A., Wilson A.M. (2020). Elevated Triglycerides to High-Density Lipoprotein Cholesterol (TG/HDL-C) Ratio Predicts Long-Term Mortality in High-Risk Patients. Heart Lung Circ..

[20] Yi S.-W., Yi J.-J., Ohrr H. (2019). Total cholesterol and all-cause mortality by sex and age: A prospective cohort study among 12.8 million adults. Sci. Rep..

[21] Petursson H., Sigurdsson J.A., Bengtsson C., Nilsen T.I.L., Getz L. (2012). Is the use of cholesterol in mortality risk algorithms in clinical guidelines valid? Ten years prospective data from the Norwegian HUNT 2 study. J. Eval. Clin. Pract..

[22] Okamura T., Tanaka H., Miyamatsu N., Hayakawa T., Kadowaki T., Kita Y., Nakamura Y., Okayama A., Ueshima H. (2007). The relationship between serum total cholesterol and all-cause or cause-specific mortality in a 17.3-year study of a Japanese cohort. Atherosclerosis.

[23] Child R.B., Wilkinson D.M., Fallowfield J.L., Donnelly A. (1998). Elevated serum antioxidant capacity and plasma malondialdehyde concentration in response to a simulated half-marathon run. Med. Sci. Sports Exerc..

[24] Orhan H., van Holland B., Krab B., Moeken J., Vermeulen N.P., Hollander P., Meerman J.H. (2004). Evaluation of a Multi-parameter Biomarker Set for Oxidative Damage in Man: Increased Urinary Excretion of Lipid, Protein and DNA Oxidation Products after One Hour of Exercise. Free Radic. Res..

[25] Dandona P., Thusu K., Cook S., Snyder B., Makowski J., Armstrong D., Nicotera T. (1996). Oxidative damage to DNA in diabetes mellitus. Lancet.

[26] Han A.L., Park S.-H., Park M.S. (2017). Hydrogen Treatment Protects against Cell Death and Senescence Induced by Oxidative Damage. J. Microbiol. Biotechnol..

[27] Sun Q., Kawamura T., Masutani K., Peng X., Sun Q., Stolz D.B., Pribis J.P., Billiar T.R., Sun X., Bermudez C.A. (2012). Oral intake of hydrogen-rich water inhibits intimal hy-perplasia in arterialized vein grafts in rats. Cardiovasc. Res..

[28] Trojanek J.B., Michalkiewicz J., Grzywa-Czuba R., Janczyk W., Gackowska L., Kubiszewska I., Helmin-Basa A., Wierzbicka-Rucińska A., Szalecki M., Socha P. (2020). Expression of Matrix Met-alloproteinases and Their Tissue Inhibitors in Peripheral Blood Leukocytes and Plasma of Children with Nonalcoholic Fatty Liver Disease. Mediat. Inflamm..

[29] Barancik M., Kura B., LeBaron T.W., Bolli R., Buday J., Slezak J. (2020). Molecular and Cellular Mechanisms Associated with Effects of Molecular Hydrogen in Cardiovascular and Central Nervous Systems. Antioxidants.

[30] Alwazeer D., Liu F.F., Wu X.Y., LeBaron T.W. (2021). Combating Oxidative Stress and Inflammation in COVID-19 by Molecular Hy-drogen Therapy: Mechanisms and Perspectives. Oxidative Med. Cell. Longev..

[31] Russell G., Rehman M., LeBaron T.W., Veal D., Adukwu E., Hancock J.T. (2020). An Overview of SARS-CoV-2 (COVID-19) Infection: The Importance of Molecular Hydrogen as an Adjunctive Therapy. React. Oxyg. Species.

[32] Slezak J., Kura B., LeBaron T.W., Singal P.K., Buday J., Barancik M. (2021). Oxidative Stress and Pathways of Molecular Hydrogen Effects in Medicine. Curr. Pharm. Des..

[33] Jackson K., Dressler N., Ben-Shushan R.S., Meerson A., LeBaron T.W., Tamir S. (2018). Effects of alkaline-electrolyzed and hydrogen-rich water, in a high-fat-diet nonalcoholic fatty liver disease mouse model. World J. Gastroenterol..

[34] Hou C., Wang Y., Zhu E., Yan C., Zhao L., Wang X., Qiu Y., Shen H., Sun X., Feng Z. (2016). Coral calcium hydride prevents hepatic steatosis in high fat diet-induced obese rats: A potent mitochondrial nutrient and phase II enzyme inducer. Biochem. Pharmacol..

[35] Ohno K., Hirayama M., Ito M., Minato T., Yoritaka A., LeBaron T.W. (2018). Inhalation of hydrogen gas elevates urinary 8-hydroxy-2′-deoxyguanine in Parkinson’s disease. Med. Gas Res..

[36] Eckermann J.M., Chen W., Jadhav V., Hsu F.P., Colohan A.R., Tang J., Zhang J.H. (2011). Hydrogen is neuroprotective against surgically induced brain injury. Med. Gas Res..

[37] Matchett G.A., Fathali N., Hasegawa Y., Jadhav V., Ostrowski R.P., Martin R.D., Dorotta I.R., Sun X., Zhang J.H. (2009). Hydrogen gas is ineffective in moderate and severe neonatal hypoxia–ischemia rat models. Brain Res..

[38] Kura B., Kalocayova B., LeBaron T.W., Frimmel K., Buday J., Surovy J., Slezak J. (2019). Regulation of microRNAs by molecular hydrogen contributes to the prevention of radiation-induced damage in the rat myocardium. Mol. Cell. Biochem..

[39] Xie Y., Mao Y., Zhang W., Lai D., Wang Q., Shen W. (2014). Reactive Oxygen Species-Dependent Nitric Oxide Production Contributes to Hydrogen-Promoted Stomatal Closure in Arabidopsis. Plant Physiol..

[40] Murakami Y., Ito M., Ohsawa I. (2017). Molecular hydrogen protects against oxidative stress-induced SH-SY5Y neuroblastoma cell death through the process of mitohormesis. PLoS ONE.

[41] LeBaron T.W., Laher I., Kura B., Slezak J. (2019). Hydrogen gas: From clinical medicine to an emerging ergogenic molecule for sports athletes. Can. J. Physiol. Pharmacol..

[42] Nishiwaki H., Ito M., Negishi S., Sobue S., Ichihara M., Ohno K. (2018). Molecular hydrogen upregulates heat shock response and col-lagen biosynthesis, and downregulates cell cycles—Meta-analyses of gene expression profiles. Free Radic. Res..

[43] Hasegawa T., Ito M., Hasegawa S., Teranishi M., Takeda K., Negishi S., Nishiwaki H., Takeda J.-I., LeBaron T.W., Ohno K. (2022). Molecular Hydrogen Enhances Proliferation of Cancer Cells That Exhibit Potent Mitochondrial Unfolded Protein Response. Int. J. Mol. Sci..

[44] Hancock J.T., LeBaron T.W., Russell G. (2021). Molecular Hydrogen: Redox Reactions and Possible Biological Interactions. React. Oxyg. Species.

