# Supplementary material for: Biological Effects of Hydrogen Water on Subjects with NAFLD: A Randomized, Placebo-Controlled Trial
Source: Antioxidants (Basel). 2022 Sep 28;11(10):1935. doi: 10.3390/antiox11101935 (PMC9598482; doi:10.3390/antiox11101935)
Supplement: Supplementary file 1 [file antioxidants-11-01935-s001.zip › antioxidants-1817817-supplementary.pdf]

Supplementary Materials

Table S1. Antibodies and dilutions used in Western blot.

Antibodies	Dilutions	Antibodies manufacturer and ID
Primary antibodies		
TNF-α, goat polyclonal	1:500	a, sc-1350
NF-κB, rabbit polyclonal	1:500	a; sc-372
HSP-70, mouse monoclonal	1:1000	a; sc-32239
HSP-60, mouse monoclonal	1:1000	a; sc-376240
SOD1, rabbit polyclonal	1:500	a; sc-11407
Secondary antibodies		
Anti-rabbit	1:2000	b; 7074S
Anti-mouse	1:2000	b; 7076S
Anti-goat	1:5000	a; sc-2020

[a] Santa Cruz biotechnology, Texas, USA; sc-1350; [b] Cell Signaling, Massachusetts, USA; 7074S
